# Clinical Factors Predicting Voluntary Driving Cessation among Patients with Parkinson's Disease

**DOI:** 10.1155/2022/4047710

**Published:** 2022-03-20

**Authors:** Hidetada Yamada, Masahiro Nakamori, Tomohisa Nezu, Teppei Kotozaki, Juri Kitamura, Tomohiko Ohshita, Yoshimasa Sueda, Hirofumi Maruyama

**Affiliations:** ^1^Department of Neurology, National Hospital Organization Higashihiroshima Medical Center, Higashihiroshima, Japan; ^2^Department of Clinical Neuroscience and Therapeutics, Hiroshima University Graduate School of Biomedical Sciences, Hiroshima, Japan

## Abstract

Factors that influence the decision of voluntary driving cessation in patients living with Parkinson's disease (PD) are still unclear. We aimed to reveal the factors affecting the decision of voluntary driving cessation in patients with PD. This hospital-based cross-sectional study recruited consecutive outpatients with PD. Data on sociodemographic and clinical characteristics and medication use were collected from the patients using semistructured interviews. Cognitive function was evaluated using the Japanese version of the Montreal Cognitive Assessment (MoCA-J). We excluded patients with dementia or motor impairment (Hoehn − Yahr stage > 3). We divided the patients into two groups, with and without voluntary driving cessation (D: driver; RD: retired driver), and conducted investigations using multivariate logistic regression analyses. Of the 40 patients, 8 (20.0%) voluntarily retired from driving. Patients who decided on driving cessation had a higher prevalence of freezing of gait (FOG) (D vs. RD, 25.0% vs. 87.5%; *P* = 0.001) and tended to have lower scores for attention in the MoCA-J (D vs. RD, 5.0 ± 1.2 vs. 4.1 ± 1.4; *P* = 0.086). Multivariable analysis showed that FOG was independently associated with driving cessation (odds ratio: 14.46, 95% confidence interval: 1.91–303.74). FOG was associated with voluntary driving cessation in patients with PD without dementia or severe motor impairment. Physicians should consider providing extensive social support to maintain patients' mobility and independence, especially if the patients have these clinical factors.

## 1. Introduction

Driving is a complex physical and cognitive task that is performed in a continually changing environment and involves numerous contributory factors [1]. In Japan, the number of fatal accidents associated with drivers aged ≥75 years is more than double that associated with drivers aged <75 years [2]. To prevent fatal traffic accidents caused by older drivers, the national and local governments in Japan provide public transport services and encourage older drivers to return their driver's licenses voluntarily [2]. However, driving cessation is associated with limited mobility and independence and can lead to health problems, such as depression, social isolation, and early death [3, 4]. Therefore, driving cessation is an important and complicated problem for the older and their families.

Parkinson's disease (PD), which is the second most common neurodegenerative disease after Alzheimer's disease, is associated with motor and nonmotor symptoms, including cognitive dysfunction [5]. In patients with PD, problems with visual perception and attention, visual and verbal memory, motor dexterity, and impairment of executive functioning have been suggested to affect driving ability [6]. Driving simulators and on-road driving assessment study have consistently shown that individuals with PD have impaired driving skills [6]. Although previous studies of driving in patients with PD have focused on driving abilities [1, 6–10], most individuals with PD cease driving based on their own decision or advice from family and/or their clinician, rather than the results of a formal driving assessment [6].

To date, there is little information available on the clinical factors that predict the patients' own decision of to voluntarily driving cessation with PD. In this study, we aimed to reveal the clinical factors that predict the patients' own decision of voluntary driving cessation, such as demographics, clinical characteristics, medications, and cognitive functions in patients with PD.

## 2. Materials and Methods

We conducted a hospital-based cross-sectional study involving consecutive outpatients with PD in the Department of Neurology at the National Hospital Organization Higashihiroshima Medical Center between July 2019 and November 2019. Our medical center is located in the central area of Hiroshima prefecture and consists of tablelands, coasts, and islands. The area of the site is approximately 797 km^2^ with a population of 227,325 and a population density of 285.2 persons/km^2^ [11]. Because of the insufficient public transport, nearly 70% of the people living in Higashihiroshima choose to drive their own cars as their principal means of transportation [12].

This study included outpatients diagnosed with PD according to the International Parkinson and Movement Disorder Society diagnostic criteria [13]. We excluded patients with a Hoehn-Yahr (H-Y) stage of >3. Patients with other clinical conditions that could significantly influence cognitive testing, such as PD with dementia, stroke, major depression, delirium, severe anxiety, and psychosis, were excluded from the International Classification of Diseases, Tenth Revision. Data on the demographics, clinical characteristics, and medication use were collected from the patients using semistructured interviews. We evaluated the motor severity and disease stage using H-Y staging. The levodopa equivalent dosage was calculated based on the formula established by Tomlinson et al. [14]. We evaluated the presence of freezing of gait (FOG) using item 3 on the Freezing of Gait Questionnaire, scored on a scale of 1–4 [15]. We evaluated sleep disorders based on the Epworth Sleepiness Scale (ESS) scores and the presence or absence of excessive daytime sleepiness [16].

We conducted an interview regarding driving and performed the Japanese version of the Montreal Cognitive Assessment (MoCA-J) in all patients. The MoCA-J was performed using the total score and six subscores, which are as follows: memory (5 points), visuospatial abilities (4 points), executive functions (4 points), attention (6 points), language (5 points), and orientation (6 points) [17, 18].

We divided the participants into two groups of patients with and without voluntary cessation of driving following the onset of PD (driver, D; retired driver, RD). The attending neurologist (T. K., J. K., Y. S.) conducted the informed consent and semistructured interviews about data on the demographics including the driving status, clinical characteristics, and medication use. The MoCA-J tests were independently conducted by another neurologist (H.Y.). The result of these interviews was analyzed blinded. Our study was performed using anonymous clinical data under close supervision following approval by the Medical Ethics Committee of the National Hospital Organization Higashihiroshima Medical Center (2019-9). Written informed consent was obtained from all patients.

### 2.1. Statistical Analysis

Data are expressed as mean ± standard deviation (SD) for continuous variables and as frequencies and percentages for discrete variables. The statistical significance of intergroup differences was assessed using the *χ*^2^ test, unpaired *t-*test, and Mann–Whitney *U* test where appropriate. First, variables with *P* < 0.10 in the univariate analysis were adjusted for age (model 1). Next, to determine the indicators for driving cessation, multivariable logistic regression models were performed using variables with *P* < 0.10 in the univariate analysis (model 2). In all analyses, a *P* value <0.05 was considered significant. All analyses were performed using JMP, Version 16.0 (SAS Institute, Inc., Cary, NC, USA).

## 3. Results

Among 50 patients with PD who regularly visited our department from July 2019 to November 2019, 40 (mean age, 72.3 ± 7.1 years; female, 55.0%; H-Y, 1.8 ± 0.6; disease duration, 5.7 ± 6.2 years) were included in the present study, and nine patients who did not provide consent were excluded. One patient who did not have a driver's license was also excluded. All the study patients were diagnosed with PD. The mean MoCA-J total score was 23.4 ± 3.0. From semistructured interviews, there was no reported cognitive decline, deficits in cognitive domains severe enough to interfere with daily living, or functional impairment based on the information gathered from standardized clinical interviews with patients and their caregivers.

The prevalence of voluntary driving cessation was 20.0%. Demographics, clinical characteristics, and MoCA-J scores were compared between patients with or without voluntary driving cessation (Table 1).

Among all clinical characteristics, patients who chose voluntary driving cessation had a longer disease duration (D vs. RD, 4.7 ± 3.3 vs. 9.6 ± 11.9; *P* = 0.042) and a higher prevalence of FOG (D vs. RD, 25.0% vs. 77.8%; *P* = 0.004). Few patients experienced FOG during driving. There was no significant difference in the total MoCA-J scores between the groups. However, patients with voluntary driving cessation tended to have lower scores for attention (D vs. RD, 5.0 ± 1.2 vs. 4.1 ± 1.4; *P* = 0.086), but this was not significant. There were no significant differences in the demographic and clinical characteristics except for the disease duration, the prevalence of FOG, and the component of attention in the MoCA-J, between the two groups. FOG was associated with driving cessation after adjustment for age (odds ratio [OR]: 35.68, 95% confidence interval [CI]: 4.42–833.32, *P* < 0.001; Table 2, model 1). However, disease duration of PD was not associated with driving cessation after adjustment for age (OR: 1.11, 95% CI: 0.92–1.21, *P* = 0.112; Table 2, model 1). Multivariable analysis revealed that FOG (OR: 14.46, 95% CI: 1.91–303.74, *P* = 0.008) was an independent factor for voluntary driving cessation (Table 2, model 2).

## 4. Discussion

In the present study, we investigated the prevalence of voluntary driving cessation among patients with PD without dementia or severe motor impairment. Of the characteristics measured, FOG was the only characteristic that predicted the patients' own decision of voluntary driving cessation in this population.

Patients with neurodegenerative diseases, including PD, may lose their autonomy and independence, even in the early stages of the disease [19]. In a previous report from the United States, owing to their demographic factors and severity of motor and cognitive function decline, drivers living with PD tended to cease driving earlier and were more likely to stop than the older control drivers [10]. Driving cessation is associated with limited mobility and independence and a poorer quality of life even in older healthy people [3, 4]. Although driving cessation in patients with PD can lead to a reduction in mobility, autonomy, and independence, 20% of the drivers with PD in the present study voluntarily ceased driving based on their own decision.

FOG is a common and disabling symptom that is experienced by 39.9% of patients with PD [20]. Freezing during driving is an important factor associated with car accidents involving drivers with PD [21]. Moreover, a recent report that focused on freezing in 6,620 patients with PD showed that 2.6% of all patients had experienced freezing at the wheel [22]. It is worth noting that people living with PD in the present study decided to cease driving voluntarily because they experienced FOG, not necessarily at the wheel. This has not been discussed in the previous studies.

Paying attention is important for safe driving [23]. A previous study revealed that poor attention was associated with near-miss traffic accidents involving older drivers [24]. In addition, in patients with neurodegenerative disorders, such as PD and Alzheimer's disease, driving errors were particularly associated with the lower performance of cognitive functions, including attention [25]. In the present study, the attention component in the MoCA-J tended to be associated with their own decision of driving cessation; however, this result was not statistically significant due to the small sample size. From the results of the present study, decline of attention might affect voluntary driving cessation among patients with PD.

A previous study showed that driving cessation was associated with increased severity of Parkinsonism and the total daily dose of antiparkinsonian medications [10]. However, a recent meta-analysis of 50 studies totaling 5,410 participants reported no evidence of an association between the degree of impairment of driving ability and disease severity, disease duration, or medication dosage [9]. In our study, the most important factor affecting the prevalence of voluntary driving cessation was FOG, regardless of disease severity. Disease duration with driving cessation was longer in the univariate analysis; however, the difference was not significant following age adjustment.

Since driving cessation is associated with limited mobility and independence and can lead to social isolation [3, 4], it is important to prevent social solitude in patients with PD following driving cessation, as well as determine the risk of driving. For such patients, interventions and initiatives that promote access to public transportation should be further implemented [26]. The local government plans to restructure and increase access to public transportation in our region [11]. Considering the aspect of machinery factors, a safe driving support car with advanced safety technology, such as automatic brakes, could support the prevention of fatal accidents and ensure safety during driving by patients with PD [2]. Furthermore, physicians should consider providing extensive social support to patients with PD and their families to maintain patients' mobility and independence, especially if the patients have these clinical factors that predict the patients' own decision of voluntary driving cessation.

There were certain limitations to our study. First, we did not assess the rate of driving accidents or patient driving ability. Hence, it is unclear whether the driving performance of patients who continued to drive was safe. A recent review reported that approximately 25% of patients with PD drive less carefully than those without PD [6]. Although there is a lack of data regarding the rate of driving accidents and driving abilities of patients with PD in the present study, results revealed that FOG played a key role in the patients' decision to voluntarily cease driving. Second, we evaluated the disease severity only using H-Y staging. Although the H-Y scale correlates with the Unified Parkinson's Disease Rating Scale (UPDRS) for disease severity, the H-Y scale could provide a limited assessment of the severity of motor symptoms or impairment of the activities of daily living compared to UPDRS [27]. Third, the living situation of the patients, such as living alone, with a partner, or with children, was not evaluated in this study. Similarly, other neighbors or caregivers were not evaluated in our study. These indicators could influence driving cessation. Fourth, this was a single-center cross-sectional study that included a small number of patients. Statistical analyses revealed large confidence intervals due to low statistical power and small sample size. In addition, multiple comparisons might have increased statistical error. Thus, for a more rigorous analysis, we analyzed multiple models in a multivariate analysis. Finally, we could not evaluate the postdriving cessation phase of the patients. Thus, we could not clarify the relationship between driving cessation and outcomes after the driving cessation of people living with PD. Prospective, large-scale, and long-term observational studies are warranted to determine whether indicators for driving cessation or other issues affect outcomes during the postdriving cessation period in patients with PD.

## 5. Conclusions

In conclusion, FOG was an important factor for voluntary driving cessation in patients with PD living in areas that are inconvenient for public transport use, regardless of disease severity. Effective interventions for improving FOG as a means to extend the driving period prior to cessation are still lacking. Physicians should pay closer attention to factors that influence patients' decision-making regarding driving and provide extensive social support to the patients to maintain their mobility and independence preventing social solitude. Further large-scale studies are warranted to determine which indicators of driving ability, including FOG, affect outcomes, and to investigate the postdriving cessation period of patients with PD.

## Figures and Tables

**Table 1:** Characteristics and scores of MoCA-J of patients with and without driving cessation.

	Total (*n* = 40)^‡^	Driver (*n* = 32, 80.0%)^‡^	Retired driver (*n* = 8, 20.0%)^‡^	*p*
Age (years)	72.3 ± 7.1	71.7 ± 7.0	74.9 ± 7.2	0.258
Female	22 (55.0)	17 (53.1)	5 (62.5)	0.634
Distance from a patient's home to a hospital (km)	11.7 ± 9.1	11.0 ± 8.8	14.6 ± 10.1	0.318
Distance from a patient's home to a railway station (km)	6.1 ± 5.3	6.2 ± 5.3	5.6 ± 5.8	0.785
Years of education (years)	13.0 ± 2.1	12.9 ± 2.2	13.4 ± 1.9	0.560
H-Y	1.8 ± 0.6	1.8 ± 0.6	2.0 ± 0.8	0.336
Disease duration (years)	5.7 ± 6.2	4.7 ± 3.3	9.6 ± 11.9	0.042
Levodopa dose (mg)	325.0 ± 153.6	334.4 ± 152.6	287.5 ± 162.0	0.447
Total anti-Parkinson drug dose^†^ (mg)	425.2 ± 209.4	415.0 ± 213.8	466.1 ± 199.0	0.544
The prevalence of freezing of gait (total)	15 (37.5)	8 (25.0)	7 (87.5)	0.001
The prevalence of freezing of gait (during driving)	1 (2.5)	1 (3.1)	0 (0.0)	0.613
The prevalence of excessive daytime sleepiness	12 (30.0)	8 (25.0)	4 (50.0)	0.168
ESS total score	6.4 ± 4.2	6.0 ± 3.5	7.8 ± 6.5	0.311
MoCA-J total score	23.4 ± 3.0	23.6 ± 3.1	22.8 ± 2.4	0.501
Memory	2.2 ± 1.6	2.2 ± 1.7	2.0 ± 1.5	0.742
Visuospatial function	3.2 ± 1.0	3.2 ± 1.1	3.3 ± 0.5	0.808
Executive function	2.9 ± 0.9	2.9 ± 0.9	2.8 ± 0.9	0.670
Attention	4.8 ± 1.2	5.0 ± 1.2	4.1 ± 1.4	0.086
Language	3.9 ± 0.9	3.8 ± 0.9	4.3 ± 0.5	0.182
Orientation	5.8 ± 0.5	5.8 ± 0.5	5.8 ± 0.5	0.872
Add one point if not educated for >12 years	0.7 ± 0.5	0.8 ± 0.4	0.6 ± 0.5	0.492

^†^ Levodopa equivalent dosage was calculated using the formula established by Tomlinson et al. [14]. ^‡^Continuous data are presented as mean ± SD, and noncontinuous data are presented as percentages. MoCA-J: the Japanese version of Montreal Cognitive Assessment; H-Y: Hoehn-Yahr stage; ESS: Epworth Sleepiness Scale.

**Table 2:** Logistic regression analysis to assess driving cessation.

Variable	Model 1		Model 2	
	OR (95% CI)	*P*	OR (95% CI)	*P*
Disease duration (per 1-year increase)	1.11 (0.92–1.21)	0.112	1.10 (0.96–1.44)	0.221
Freezing of gait (freezing of gait was present)	35.68 (4.42–833.32)	<0.001	14.46 (1.91–303.74)	0.008
Attention (per 1 point increase)	0.58 (0.29–1.09)	0.090	0.75 (0.35–1.53)	0.427

Model 1: each variable is adjusted for age. Model 2: multivariate analysis was performed by variables with *P* < 0.10 in the univariate analysis. OR: odds ratio; CI: confidence interval.

## Data Availability

The datasets used and/or analyzed during the current study are available from the corresponding author upon reasonable request.
